# To beat or not to beat a tick: comparison of DNA extraction methods for ticks (*Ixodes scapularis*)

**DOI:** 10.7717/peerj.1147

**Published:** 2015-08-13

**Authors:** Alyssa D. Ammazzalorso, Christine P. Zolnik, Thomas J. Daniels, Sergios-Orestis Kolokotronis

**Affiliations:** 1 Department of Biological Sciences, Fordham University, Bronx, NY, USA; 2 Vector Ecology Laboratory, Louis Calder Center–Biological Field Station, Fordham University, Armonk, NY, USA

**Keywords:** Arthropod, Vector-borne, Blacklegged tick, Tick, DNA extraction, Nucleic acids, DNA quantification

## Abstract

**Background.** Blacklegged ticks (*Ixodes scapularis*) are important disease vectors in the United States, known to transmit a variety of pathogens to humans, including bacteria, protozoa, and viruses. Their importance as a disease vector necessitates reliable and comparable methods for extracting microbial DNA from ticks. Furthermore, to explore the population genetics or genomics of this tick, appropriate DNA extraction techniques are needed for both the vector and its microbes. Although a few studies have investigated different methods of DNA isolation from ticks, they are limited in the number and types of DNA extraction and lack species-specific quantification of DNA yield.

**Methods.** Here we determined the most efficient and consistent method of DNA extraction from two different developmental stages of *I. scapularis*—nymph and adult—that are the most important for disease transmission. We used various methods of physical disruption of the hard, chitinous exoskeleton, as well as commercial and non-commercial DNA isolation kits. To gauge the effectiveness of these methods, we quantified the DNA yield and confirmed the DNA quality via PCR of both tick and microbial genetic material.

**Results.** DNA extraction using the Thermo GeneJET Genomic DNA Purification Kit resulted in the highest DNA yields and the most consistent PCR amplification when combined with either cutting or bead beating with select matrices across life stages. DNA isolation methods using ammonium hydroxide as well as the MoBio PowerSoil kit also produced strong and successful PCR amplification, but only for females.

**Discussion.** We contrasted a variety of readily available methods of DNA extraction from single individual blacklegged ticks and presented the results through a quantitative and qualitative assessment.

## Introduction

Blacklegged ticks (*Ixodes scapularis* Say, 1821) are hard-bodied, hematophagous arthropod (Arachnida, Ixodida) ectoparasites of vertebrates in North America. During its two-year life cycle, the tick acquires a bloodmeal at each developmental stage (i.e., larva, nymph, and adult) prior to molting or egg-laying, in the case of adult females. These ticks are of great public health importance as pathogen vectors because they carry and transmit a variety of human disease agents, such as *Borrelia burdgorferi*, the causative agent of Lyme disease, *Anaplasma phagocytophilum* which causes human granulocytic anaplasmosis, and *Babesia microti*, a protozoan responsible for the malaria-like illness, babesiosis (Speilman, 1976; Steere, Broderick & Malawista, 1978; Pancholi et al., 1995). Recently, *I. scapularis* has been found to transmit *Borrelia miyamotoi* (Scoles et al., 2001) and Powassan virus lineage 2 (a.k.a., Deer Tick Virus) (Telford et al., 1997). Their importance as human pathogen vectors necessitates research that involves successful isolation of genetic material needed in investigations of both the vector itself and of the wide range of pathogens that they carry. However, DNA isolation in ticks is challenging due to the hard chitinous exoskeleton and the small amount of microbial nucleic acids present (Halos et al., 2004). Furthermore, tick DNA is suseptible to degradation (Hubbard, Cann & Wright, 1995; Hill & Gutierrez, 2003; Halos et al., 2004) and PCR can be challenged by inhibitors (Halos et al., 2004).

Although DNA extraction from ticks for both pathogen isolation and tick genetic and genomic research is performed routinely by researchers, there is no consensus regarding the most effective method of DNA isolation from any tick species. A few such studies and reviews have been conducted (Hill & Gutierrez, 2003; Halos et al., 2004; Crowder et al., 2010; Mtambo et al., 2006; Sparagano et al., 1999); however, they are limited to a handful of extraction techniques, and quantitative data on DNA concentration is lacking. In this study we aim to identify the optimal DNA isolation procedure for both tick and microbial DNA from an important tick pathogen vector, the blacklegged tick.

## Materials & Methods

### Tick collection

Nymphal and adult female blacklegged ticks are important life stages in the transmission of disease agents compared to larvae, which are rarely infected with human pathogens, and adult males, whose brief feeding bouts minimize the risk of pathogen transmission (Piesman et al., 1986; Falco & Fish, 1988; Falco et al., 1999). Thus, the ability to dependably extract DNA, and in particular microbial DNA, from nymphal and adult female blacklegged ticks is of importance to tick-borne disease research and constitutes the focus of this study.

Unfed, host-seeking nymphal and adult female blacklegged ticks were collected by dragging a 1 m^2^ flannel cloth along the forest floor or along low vegetation, respectively, during each life stage’s peak activity period. A total of 258 ticks (129 nymphs and 129 adult females) were collected from sites in Westchester County, Putnam County, and Orange County in New York state in the summers and falls of 2009–2011, and were subsequently stored in 70% v/v ethanol at room temperature until DNA isolation in the summer of 2014.

### DNA isolation

We contrasted the efficiency of extracting DNA from ticks stored in 70% v/v ethanol using five different DNA isolation procedures coupled with either cross-sectional division or bead-based physical disruption of the tick body. These procedures included four commercially available DNA extraction kits: DNeasy Blood & Tissue Kit (QIAGEN, Valencia, California, USA), GeneJET Genomic DNA Purification Kit (Thermo Scientific, Waltham, Massachusetts, USA), Tissue & Insect DNA MicroPrep (Zymo Research, Irvine, California, USA), PowerSoil DNA Isolation Kit (MoBio, Carlsbad, California, USA); and one noncommercial DNA extraction method using ammonium hydroxide (NH_4_OH) and heat, which has been primarily used for DNA isolation from the European sheep tick, *Ixodes ricinus* (Guy & Stanek, 1991; Rijpkema et al., 1996; Pichon et al., 2003; Humair et al., 2007; Pangrácová et al., 2013).

All commercial kits examined here use a silica-based column procedure and have either been used in previous studies for DNA isolation in ticks or are marketed for efficient microbial DNA recovery or insect DNA isolation (Table 1). All kits also include filtering through a silica gel membrane and a variety of associated buffers. The Zymo kit and the NH_4_OH treatment did not include a protein digestion step. DNA extraction kits were used according to the manufacturers’ recommended protocols with few exceptions (Table 1), including a final elution completed following a 5-min room-temperature incubation in 100 µl of deionized, sterilized, distilled water (sdH_2_O) for consistency. Each tick was air-dried to evaporate the ethanol prior to DNA extraction, as ethanol may inhibit PCR (Hubbard, Cann & Wright, 1995; Bessetti, 2007; Schrader et al., 2012). Due to the components similarity of the QIAGEN and Thermo kits, we used the same Proteinase-K incubation duration (overnight).

**Table 1:** Methods of DNA isolation and physical disruption of tick samples. Only samples treated with the MoBio kit were processed on the GeneMate vortex mixer (BioExpress), while all remaining bead beating took place on the BeadBlaster 24 (Benchmark Scientific).

DNA extraction method	Alterations to manufacturer protocols	Physical disruption	Bead beating —speed and duration
**QIAGEN DNeasy Blood & Tissue Kit** (cat. no. 69506)	Overnight incubation at 56 °C in lysis buffer/proteinase K	Bisection (Nymphs), Quadrisection (Females)	N/A
	Elution in 100 μl dsH_2_0 with 5 min room temperature incubation	MP Bio Lysing Matrices	4 m/s 1.5 & 4.0 min
**Thermo GeneJET Genomic DNA Purification Kit** (cat. no. K0722)	Overnight incubation at 56 °C in lysis buffer/proteinase K	Bisection (Nymphs), Quadrisection (Females)	N/A
	Elution in 100 μl dsH_2_0 with 5 min room temperature incubation	MP Bio Lysing Matrices	4 m/s 1.5 & 4.0 min
**Zymo Research Tissue & Insect DNA MicroPrep** (cat. no. D6015)	Elution in 100 μl dsH_2_0 with 5 min room temperature incubation	Bisection (Nymphs), Quadrisection (Females) followed by beating with Zymo beads	4 m/s 10 min
		Zymo beads	4 m/s 10 min
**MoBio PowerSoil DNA Isolation Kit** (cat. no. 12888)	Elution in 100 μl of dsH_2_0 with 5 min room temperature incubation	Bisection (Nymphs), Quadrisection (Females) followed by vortexing with MoBio garnet beads	3,200 rpm 10 min
		MoBio provided beads	3,200 rpm 10 min
**NH_4_OH** (Guy & Stanek, 1991; Pichon et al., 2003)	Initial volume of 150 μl NH_4_OH Final volume of 70–100 μl dsH20	Bisection (Nymphs), Quadrisection (Females)	N/A
	Second centrifugation for 2 min at 10,000 × *g*	MP Bio Lysing Matrices	4 m/s 1.5 & 4.0 min

The NH_4_OH method included adding 150 µl of a 0.7-M NH_4_OH solution to the tick sample in a 1.5-ml snap cap tube, and heating to 100 °C for 15 min. The solution was briefly centrifuged to concentrate fluid at the bottom and then was evaporated to 70–100 µl by opening the tubes and heating at 100 °C for an additional 15 min. The solution was then centrifuged for 10 min at 10,000 × *g* and the supernatant was collected and respun for 2 min at 10,000 × *g*. The total supernatant was collected and stored at −20 °C.

To determine the most effective method of physical disruption of the hard, chitinous exoskeleton of ticks prior to DNA extraction, we compared cross-sectionally dividing ticks (bisection for nymphs and quadrisection for adult females) and crushing the entire tick in a variety of bead matrices using the BeadBlaster 24 (Benchmark Scientific, Edison, New Jersey, USA) (Table 2). The MoBio and Zymo kits included their own bead matrices (Table 2). The beads from these two kits were used according to the manufacturers’ instructions with either whole or cut ticks. MoBio-processed samples were beaten on a GeneMate vortex mixer (BioExpress, Kaysville, Utah, USA) at maximum speed (3,200 rpm) for 10 min, and Zymo-processed samples were beaten on the BeadBlaster 24 for 10 min at 4 m/s. For DNA extraction methods that did not include bead matrices (i.e., QIAGEN, Thermo, and NH_4_OH), we used six different MP Bio Lysing Matrices (http://www.mpbio.com/index.php?cPath=2_77_425&country=223), which were either marketed for tough samples or, as in the case of Matrices H and I, were marketed specifically for ticks (Table 2). We beat the ticks with the MP Bio Lysing Matrices for 1.5 and 4 min at 4 m/s (Table 1).

**Table 2:** Bead matrices and their attributes. The composition, characteristics, and recommended uses for the different bead matrices tested are adapted from the manufacturers’ websites. (MP Bio: http://www.mpbio.com/index.php?cPath=2_77_425&country=223, MoBio: http://www.mobio.com/soil-dna-isolation/powersoil-dna-isolation-kit.html, Zymo: http://www.zymoresearch.com/dna/genomic-dna/solid-ffpe-tissue-dna/zr-tissue-insect-dna-miniprep).

Matrix	Manufacturer	Material	Suggested use
**G**	MP Bio	1.6 mm silicon carbide particles	Samples with tough, hard, or brittle cell membranes
**H**	MP Bio	2 mm glass beads & 2 mm zirconium oxide beads	Tough, hard cells and organisms within dense exterior matrices, e.g., whole insects and ticks
**I**	MP Bio	2 mm zirconium beads & one 4 mm ceramic sphere	Very tough, hard samples including chitin exoskeletons, e.g., whole insects and ticks
**M**	MP Bio	Two 6.35 mm zirconium oxide-coated ceramic grinding spheres	Tough tissues, seeds, spores
**S**	MP Bio	3.175 mm stainless steel beads	Tough tissues, seeds, spores
**Z**	MP Bio	2.0 mm yttria-stabilized zirconium oxide spheres	Tough plant and animal samples
**PowerBeads**	MoBio	Garnet	Environmental samples
**BashingBead Lysis Matrix**	Zymo	Ceramic	Ticks, mosquitoes, bees, lice, and *Drosophila melanogaster*

The variables that we maintained constant across DNA extraction methods were: bead beating speed (4 m/s), water volume for final DNA elution of 100 µl, and a 5-min duration of incubation in sdH_2_O during DNA elution.

DNA was extracted from three nymphs and three females for all combinations of DNA isolation and physical disruption procedures, including the different bead beating durations and MP Bio Lysing Matrices. DNA was extracted using the MoBio and Zymo kits from six nymphs and six females each. Half were bisected or quadrisected and half remained whole prior to DNA extraction with one of these two kits. The QIAGEN, Thermo, and NH_4_OH methods were used to extract DNA from 39 nymphs and 39 females each. For every one of these three procedures, three ticks from each life stage were bisected or quadrisected and 36 ticks were bead-beaten. Among the 36 bead-beaten ticks processed per method, six nymphs and six females were beaten with one of the six MP Bio Lysing Matrices, half for 1.5 min and half for 4 min. Overall, DNA was isolated from 129 nymphs and 129 females for a total of 258 tick DNA extractions.

### DNA quantification

The resulting DNA yields were quantified via double-stranded DNA (dsDNA) fluorometric quantitation on a Qubit 2.0 flurometer (Life Technologies, Norwalk, Connecticut, USA) using 10 µl of extracted DNA template in 190 µl of the High Sensitivty (HS) dsDNA assay.

### PCR validation

The isolated DNA was validated using PCR amplification of tick mitochondrial and nuclear loci. We amplified the tick DNA using (1) the cytochrome *c* oxidase subunit 1 (*Cox1*) DNA barcode region (∼650 bp) located on the mitochondrial genome with the HCO/LCO primers (Folmer et al., 1994), and (2) a dinucleotide (CA)_*n*_ microsatellite repeat located on the nuclear genome with the bac7ea/bac7eb primer pair (139–197 bp) (Chan, 2012).

We targeted the genus *Rickettsia* using PCR to validate the successful extraction of microbial DNA from inside the tick, which is necessary for studies involving PCR detection of human pathogens transmitted by this tick species. Members of this genus are obligate intracellular bacteria and are abundant in blacklegged ticks (Benson et al., 2004; Moreno et al., 2006; Noda, Munderloh & Kurtti, 1997; Steiner et al., 2008). We targeted a 532-bp fragment of the *ompA* gene using *Rickettsia*-specific primers (Vitorino et al., 2007).

All thermal cycling conditions were slightly modified from their published protocols and are detailed below. The thermal cycling conditions for the *Cox1* DNA barcode region began with an initial denaturation at 95 °C for 5 min, followed by 35 cycles of 95 °C for 30 s, 50 °C for 30 s, and 72 °C for 30 s, and then a final extension at 72 °C for 7 min (Folmer et al., 1994). The microsatellite region was amplified using a touchdown PCR with an initial denaturation at 95 °C for 1 min, then 5 cycles of 95 °C for 20 s, 60 °C for 20 s, and 72 °C for 30 s; 30 cycles of 95 °C for 20 s, 50 °C for 25 s, and 72 °C for 30 s; and a final extension for 5 min at 72 °C (Chan, 2012). The *Rickettsia ompA* locus was amplified with an initial denaturation at 95 °C for 5 min, 35 cycles of 95 °C for 30 s, 52 °C for 30 s, and 72 °C for 30 s, and a final extension for 7 min at 72 °C (Vitorino et al., 2007). All PCR reactions were performed using a Techne Prime Elite Thermal Cycler (Bibby Scientific, Burlington, New Jersey, USA).

Each PCR was performed in a final 25-µl volume with 6.25 µl 2 × MyTaq HS Mix (Bioline, Taunton, Massachusetts, USA) and 0.2 µM of each forward and reverse primer. The PCRs targeting nuclear and mitochondrial tick DNA were caried out using 16.25 µl sdH_2_O and 1.5 µl DNA template. To account for the lower DNA concentrations of microbial DNA within this tick, and specifically for nymphs, the PCRs targeting the *Rickettsia ompA* fragment were carried out with 15.75 µl sdH_2_O and 2 µl DNA template from nymphs, and 16.75 µl sdH_2_O and 1 µl DNA template for adult females. PCRs were confirmed by agarose gel electrophoresis on a 1.5% w/v gel based on amplicon length.

### Statistical analysis

Given the substantial differences in size and DNA yield between nymphs and adult females, their DNA concentrations were not comparable. Therefore, statistical analyses of the DNA concentration data from the two developmental stages were performed separately. Following a one-way ANOVA, Tukey’s HSD test was used for post-hoc analysis of the average DNA quantification values resulting from nymph bisection or female quadrisection across the five DNA isolation methods (QIAGEN, Thermo, MoBio, Zymo, and NH_4_OH). The Bonferroni correction was used to account for multiple comparisons. The same procedures were also used to compare the DNA yields resulting from the different MP Bio Lysing Matrices (G, H, I, M, S, and Z) and nymph bisection or female quadrisection methods using data from the highest-yielding DNA extraction method. The effect of bead beating duration was assessed through a two-tailed Student’s *t*-test.

## Results and Discussion

### Comparison of DNA yield based on bisection and quadrisection

**Figure 1:**
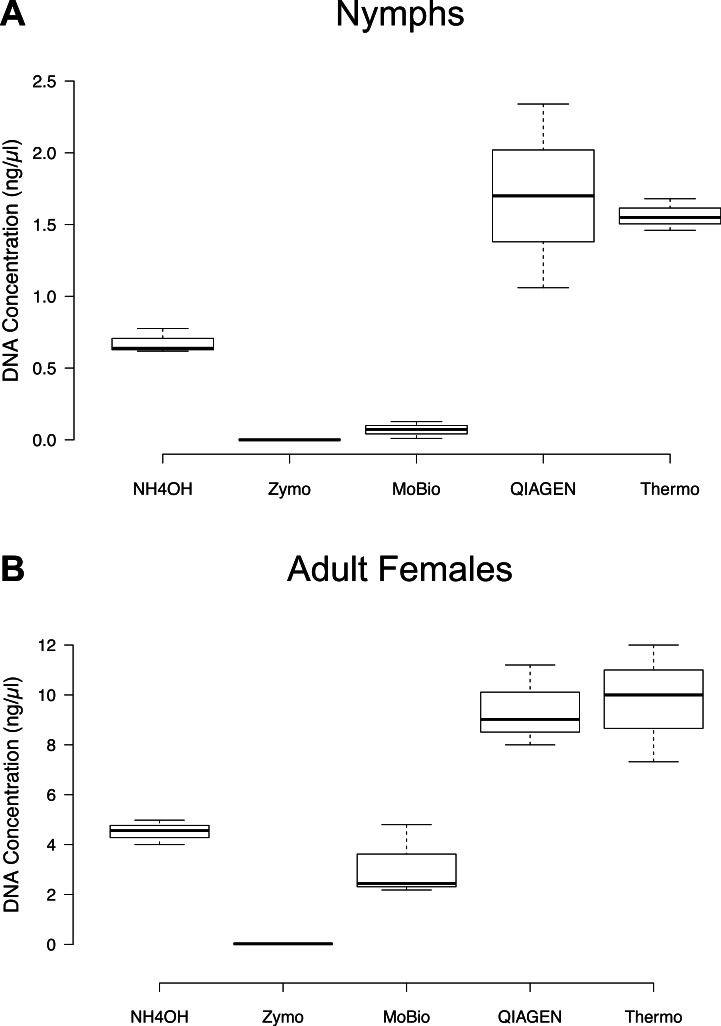
DNA concentrations (ng/µl) resulting from the five DNA extraction methods following nymph bisection and female quadrisection as determined using the Qubit HS dsDNA Assay. Each sample set consisted of three individual tick DNA extractions. Note the difference in scale between the life stages. (A) Nymphs (B) Adult Females.

The comparison of DNA concentrations resulting from nymph bisection and female quadrisection across the five DNA extraction methods yielded no apparent differences between the QIAGEN DNEasy Blood & Tissue Kit and the Thermo GeneJET Genomic DNA Purification Kit (Fig. 1). For both nymphs and adult females, the QIAGEN and Thermo methods resulted in markedly higher DNA yields than all other methods (Fig. 1, Table 3). The Zymo Research Tissue & Insect DNA MicroPrep Kit produced DNA yields that were too low to be quantifiable by the Qubit fluorometer in the case of nymphs and were extremely low in the case of adult females (average = 0.02 ng/µl, SD = 0.01).

**Table 3:** Average DNA concentration (ng/μl) of whole and cut nymphal and adult female blacklegged ticks. Average and standard deviation of the DNA concentration values determined using the Qubit HS dsDNA Assay. Unless otherwise indicated, samples were stored in 70% v/v ethanol. Three single-tick measurements were included in each treatment. All values listed as <0.0005 ng/μl indicate a reading of “too low” from the Qubit fluorometer.

Method	Life stage	Nymphs		Adult females	
		Bisected	Whole	Quadrisected	Whole
QIAGEN	Average	1.70	Table S1	9.41	Table S1
	SD	0.640		1.63	
Thermo	Average	1.68	Table S1	9.77	Table S1
	SD	0.11		2.35	
MoBio	Average	0.070	0.0373	4.51	0.119
	SD	0.0585	0.0457	0.492	0.0133
Zymo	Average	<0.0005	<0.0005	3.14	0.0146
	SD	0.00	0.00	1.44	0.00341
NH_4_OH	Average	0.677	Table S1	0.0240	Table S1
	SD	0.086		0.0143	

### Comparison of DNA yield based on bead beating

**Figure 2:**
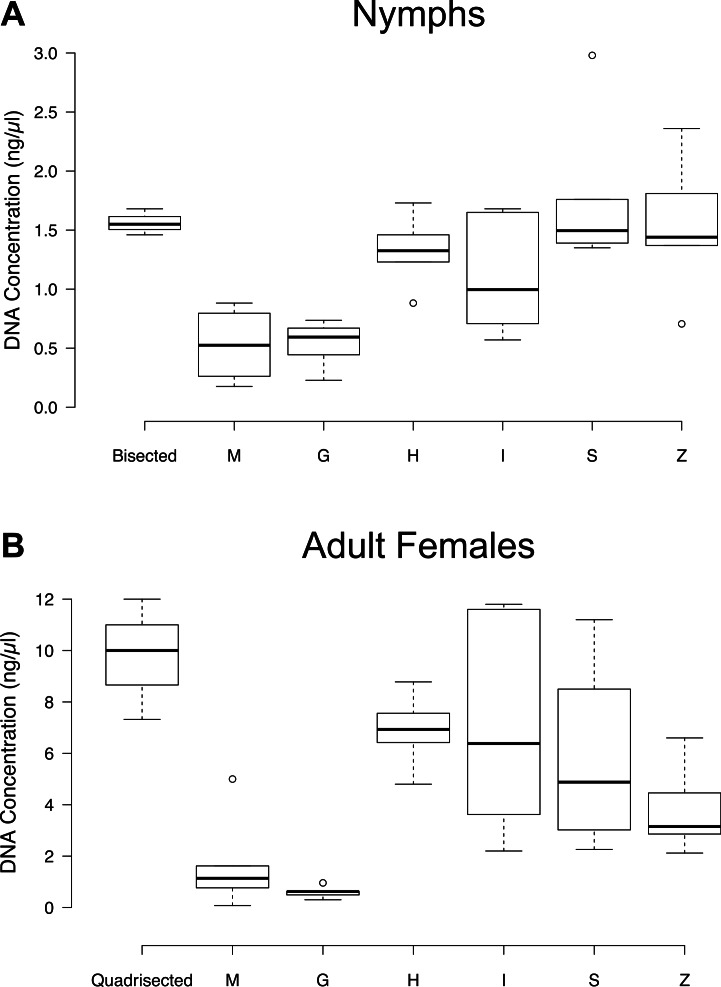
DNA concentrations (ng/µl) resulting from the Thermo DNA extraction method following the bead beating of whole ticks. Bead beating was carried out with each of the MP Bio lysing matrices (G, H, I, M, S, and Z). Nymphs were bisected and adult females were quadrisected. The DNA concentration was determined using the Qubit HS dsDNA Assay. Six nymphs and six adults were used in each bead beating treatment, while three nymphs and three adults were used in each cutting treatment. (A) Nymphs (B) Adult Females.

For the two highest-yielding DNA isolation methods (QIAGEN and Thermo), Thermo had a significantly higher DNA yield than QIAGEN across all bead matrices in the case of both nymphs (*P* = 2.7e–06) and females (*P* = 0.02). When cut ticks were included in this comparison, Thermo had a higher DNA yield for both nymphs (*P* = 2.39e–05) and females (*P* = 0.04). Therefore, in this section we report results based solely on the Thermo method. Specifically, the use of bead matrices H, I, S, and Z for nymphs, and H, I, and S for females resulted in higher DNA yield (*P* < 0.04). For both nymphs and adult females the M and G matrices were the least efficient (Fig. 2). Nymphal bisection also produced DNA yields that were significantly better than M and G, while adult female quadrisection outperformed matrices M, G, and Z (*P* < 0.03) (Fig. 2). The duration of bead beating had no effect on DNA yield in either nymphs or adult females (*P* > 0.6 and *P* > 0.1, respectively) MP Bio lysing matrix bead beating results for whole nymphs and adult females treated with the QIAGEN, Thermo and NH_4_OH methods are detailled in Table S1.

### PCR validation

The Thermo method exhibited the strongest and most consistent gel electrophoresis PCR product bands across physical disruption methods in the case of both nymphs and females for mtDNA (*Cox1*), nuDNA (microsatellite), and bacterial DNA (*Rickettsia ompA*). The DNA extractions resulting from the NH_4_OH protocol yielded strong and mostly consistent amplification of the tick mitochondrial and nuclear loci, and the bacterial gene for adult females. Although the NH_4_OH method yielded lower DNA concentration results than the QIAGEN and Thermo kits when using bead-beaten or quadrisected adult female ticks, this did not affect PCR success. However, PCR amplification was consistently poor for nymphs treated with the NH_4_OH protocol. Consistent with the very low DNA yields we measured, the DNA extracted using the Zymo kit did not result in any successful PCRs for ticks (either nymphs or adults) and only produced amplicons for *Rickettsia ompA* from quadrisected females.

DNA extraction from adult female ticks with the MoBio kit resulted in the consistent amplification of the three targeted loci, with quadrisection substantially enhancing the amplicon gel band quality in contrast to whole females (beaten with garnet on a vortex). Those loci were not successfully amplified in the case of bisected and whole nymphs—concordant with low DNA yields.

Among the MP Bio Lysing Matrices H, I, and S produced some of the strongest and most consistent PCR results across all targeted loci following bead-beating for 1.5 min for both females and nymphs with the Thermo kit and for females with the NH_4_OH method. These results partially reflected the MP Bio website’s recommendation for using matrices H and I with ticks, while matrix S is not mentioned in that capacity.

Nymph bisection and female quadrisection produced PCR products that were generally just as strong (on agarose gel) and consistent as bead beating with the H, I, and S matrices combined with the Thermo kit. In the case of NH_4_OH only adult females (quadrisected and bead-beaten with matrices H, I, and S) had successful PCR results. The QIAGEN kit consistently yielded successful PCR amplification when ticks were cut, but when using bead beating PCR results were mixed and inconsistent.

In terms of rapidly quantifying PCR success, we can report the proportion of positive PCRs, while acknowledging that this is merely based on PCR product band presence on an agarose gel, rather than accounting for band intensity. The latter characteristic can be discussed on case-by-case basis, rather than being summarized in subjective fluorescence categories, e.g., faint, medium, strong. In terms of DNA extraction methods, the Thermo kit yielded the most “amplifiable” DNA (201 positive PCRs out of 234, i.e., 85.9% success rate) with comparable performance across developmental stages (87.18% of adult females vs. 84.62% of nymphs). The two other methods that used bead beating (QIAGEN and NH_4_OH) did not perform as well overall (61.11% and 58.55%, respectively) and furthermore exhibited a skewed behavior across developmental stage with PCRs from female adult ticks working more consistently (72.65% of females vs. 49.57% of nymphs for QIAGEN; 85.47% of females vs. 31.62% of nymphs in NH_4_OH). PCRs using the DNA extracted with the MoBio kit worked well with female adults (88.89%) but not with nymphs (11.11%). The Zymo kit was the most poorly performing in terms of PCR with only three successful reactions out of 36 (8.33%) with none of the nymphal samples producing amplified PCR products. Those DNA isolation and physical disruption methods that were most successful for the nuclear and mitochondrial tick gene PCRs were also most effective for the *Rickettsia ompA* gene amplification. All of this information is detailed in Table S2, yet we raise caution against the strict interpretation of those results, as part of a full assessment should be the PCR amplicon yield (i.e., number of PCR fragments) that, during gel electrophoresis, is manifested by fluorescence intensity. Quantitative PCR can be used for such purposes.

## Conclusions

Successful DNA extraction from tick species is important for both genetic and genomic studies of the tick vector itself, as well as for studies aimed at detecting pathogen presence in these tick vectors. This study was designed to determine the most reliable and efficient method of DNA extraction, including physical disruption of the tick exoskeleton.

Among the tested tick DNA extraction procedures, we recommend several different procedures depending on budget, time, contamination concerns, and study goals. The Thermo kit is recommended for its high DNA yields coupled with high-quality PCR amplification with both bead beating and nymph bisection or adult quadrisection, as well as for its lower cost in comparison with the QIAGEN kit—a similarly structured kit. The QIAGEN kit may be used if already available when cutting ticks, but is not recommended if bead beating is the chosen physical disruption strategy.

The NH_4_OH extraction method is an inexpensive alternative to commercially available kits and produced high-quality PCR products for adult females, although the DNA yield was generally lower than that of commercial kits. However, this method was not useful for DNA extraction from nymphs, resulting in low DNA yield and poor to non-existent PCR amplification, despite the frequent use of this method in studies on nymphal *Ixodes ricinus* in Europe (Guy & Stanek, 1991; Rijpkema et al., 1996; Pichon et al., 2003; Humair et al., 2007; Pangrácová et al., 2013).

The MP Bio H and I matrices, which MP Bio recommends for ticks, as well as the S matrix were best for bead beating in conjunction with the Thermo kit for nymphs and females or NH_4_OH for females. These results confirm MP Bio’s recommendation of the H and I matrices for ticks, and we would add to that list matrix S. Benefits of bead beating include less processing time and reduced direct sample handling, which may decrease the likelihood of contamination. However, cutting ticks requires less expensive equipment than bead beating.

The Zymo kit was a poor choice for extracting DNA from nymphs and adult female ticks, although it is marketed for DNA extraction from stored arthropods, including ticks (as per the manufacturer’s website http://www.zymoresearch.com/dna/genomic-dna/solid-ffpe-tissue-dna/zr-tissue-insect-dna-kits/zr-tissue-insect-dna-microprep). The MoBio kit also performed poorly with nymphs but showed suceess with adult females.

Admittedly, the sample size of three nymph and three female replicates for each combination of DNA extraction variables is limited. Additional replicates may better capture the potential variability in DNA concentration and quality resulting from each extraction procedure. Future studies with larger sample sizes would be useful to further assess the efficacy of these different methods with perhaps greater precision, although there is no reason to expect that increasing the number of replicates would necessarily yield globally different results. Our results clearly demonstrate that DNA yield quality varies among different extraction kits and methods, which can have an important impact on the success of PCR-based studies.

Our study expands on previous work that determined DNA extraction success from ticks based on PCR amplification alone, without a DNA quantification assessment (Halos et al., 2004). While a recent study (Crowder et al., 2010) quantified DNA yield, the reported values were averaged across multiple tick species and focused only on one developmental stage—adults. In order to test the efficiency of the DNA extraction techniques, we kept certain variables constant, such as the long-term storage method, bead beating speed, elution volume, and incubation time prior to elution. Alteration of these variables may result in increased DNA yield and should be considered when DNA concentration is important in downstream applications, such as high-throughput sequencing, pathogen surveillance, and microbial community profiling.

## Supplemental Information

10.7717/peerj.1147/supp-1 Table S1 Average DNA concentration (ng/µl) of whole nymphal and adult female blacklegged ticks bead-beaten with MP Bio lysing matrices The results of DNA extractions from whole ticks after bead beating for QIAGEN, Thermo, and NH4OH (see Table 3) are expanded here to include the individual results from each of the six MP Bio Lysing Matrices. Average and standard deviation of the DNA concentration values were determined using the Qubit HS dsDNA Assay (*n* = 3).

10.7717/peerj.1147/supp-2 Table S2 PCR amplification success for each DNA extraction method and physical disruption type
