# Supplementary material for: To beat or not to beat a tick: comparison of DNA extraction methods for ticks (Ixodes scapularis)
Source: PeerJ. 2015 Aug 13;3:e1147. doi: 10.7717/peerj.1147 (PMC4540005; doi:10.7717/peerj.1147)
Supplement: Table S1 — The results of DNA extractions from whole ticks after bead beating for QIAGEN, Thermo, and NH4OH (see Table 3) are expanded here to include the individual results from each of the six MP Bio Lysing Matrices. Average and standard deviation of the DNA concentration values were determined using the Qubit HS dsDNA Assay (n = 3). [file peerj-03-1147-s001.docx]

**Table S1. Average DNA concentration (ng/μl) of whole nymphal and adult female blacklegged ticks bead-beaten with MP Bio lysing matrices.** The results of DNA extractions from whole ticks after bead beating for QIAGEN, Thermo, and NH_4_OH (see Table 3) are expanded here to include the individual results from each of the six MP Bio Lysing Matrices. Average and standard deviation of the DNA concentration values were determined using the Qubit HS dsDNA Assay (n=3).

| **Method** | **Life Stage** | **Nymphs** | | | | | | **Adult Females** | | | | | |
| --- | --- | --- | --- | --- | --- | --- | --- | --- | --- | --- | --- | --- | --- |
|  | **Matrix** | **G** | **H** | **I** | **M** | **S** | **Z** | **G** | **H** | **I** | **M** | **S** | **Z** |
| QIAGEN  1.5 min | Average | 0.725 | 0.398 | 0.464 | 0.364 | 0.995 | 0.337 | 2.995 | 2.915 | 1.051 | 7.147 | 2.460 | 2.237 |
|  | SD | 0.062 | 0.065 | 0.336 | 0.287 | 0.709 | 0.260 | 1.824 | 2.509 | 0.302 | 3.933 | 1.963 | 1.116 |
| QIAGEN  4 min | Average | 0.634 | 0.545 | 0.674 | 0.290 | 1.078 | 0.393 | 0.889 | 1.001 | 2.293 | 2.589 | 1.065 | 4.208 |
|  | SD | 0.044 | 0.244 | 0.153 | 0.024 | 0.229 | 0.180 | 0.226 | 0.294 | 1.903 | 1.387 | 0.430 | 3.864 |
| Thermo  1.5 min | Average | 0.647 | 1.281 | 1.493 | 0.341 | 1.540 | 1.195 | 0.629 | 6.080 | 3.220 | 2.710 | 3.113 | 4.300 |
|  | SD | 0.102 | 0.426 | 0.298 | 0.215 | 0.195 | 0.430 | 0.329 | 1.148 | 0.890 | 1.984 | 0.904 | 1.993 |
| Thermo  4 min | Average | 0.441 | 1.370 | 0.707 | 0.715 | 1.950 | 1.847 | 0.579 | 7.727 | 10.773 | 0.537 | 8.453 | 3.147 |
|  | SD | 0.212 | 0.085 | 0.136 | 0.220 | 0.896 | 0.496 | 0.075 | 0.981 | 1.608 | 0.399 | 2.770 | 1.196 |
| NH_4_OH  1.5 min | Average | 0.256 | 0.223 | 0.219 | 0.527 | 0.209 | 0.367 | 0.841 | 2.067 | 1.213 | 2.760 | 2.777 | 1.763 |
|  | SD | 0.012 | 0.094 | 0.072 | 0.027 | 0.036 | 0.104 | 0.290 | 1.139 | 0.154 | 0.246 | 0.817 | 0.161 |
| NH_4_OH  4 min | Average | 0.362 | 0.309 | 0.197 | 0.398 | 0.252 | 0.463 | 0.673 | 0.423 | 1.173 | 2.527 | 0.771 | 1.220 |
|  | SD | 0.034 | 0.033 | 0.080 | 0.023 | 0.091 | 0.031 | 0.234 | 0.138 | 0.176 | 0.794 | 0.188 | 0.095 |
